# Profiling of Differentially Expressed MicroRNAs in Saliva of Parkinson's Disease Patients

**DOI:** 10.3389/fneur.2021.738530

**Published:** 2021-11-26

**Authors:** Yanyan Jiang, Jing Chen, Yunchuang Sun, Fan Li, Luhua Wei, Wei Sun, Jianwen Deng, Yun Yuan, Zhaoxia Wang

**Affiliations:** ^1^Department of Neurology, Peking University First Hospital, Beijing, China; ^2^Key Laboratory of Neurovascular Disease Discovery, Beijing, China

**Keywords:** Parkinson's disease, diagnosis, saliva, biomarker, microRNA

## Abstract

**Objective:** This study aims to identify differentially expressed salivary miRNAs and validate the diagnostic potential for idiopathic Parkinson's disease (PD). Also, the disease specificity of candidate miRNAs was evaluated between PD, multiple system atrophy (MSA), and essential tremor (ET).

**Methods:** We collected salivary samples from 50 PD, 20 ET, and 20 MSA patients, as well as 30 healthy controls (HCs). In the discovery phase, salivary miRNA microarray analysis was performed. *In-silico* analysis was used to investigate the target genes of differentially expressed miRNAs and clustered pathways. In validation phase, RT-qPCR was performed with samples from 30 PD patients and 30 HCs. Subsequently, we investigated candidate miRNAs in all recruited subjects. Receiver operating characteristic curve and Spearman correlation analysis was performed to determine diagnostic usefulness.

**Results:** We identified 43 miRNAs that were differentially expressed between 5 PD patients and 5 HCs by miRNA microarray analysis. Computational analysis revealed the target genes were clustered in the pathways associated with ubiquitin protein ligase activity. The result of RT-qPCR showed that the miR-29a-3p and miR-29c-3p were found to be significantly downregulated (*p* = 0.004, *p* = 0.027), whereas the miR-6756-5p was significantly upregulated in 30 PD patients compared with 30 HCs (*p* = 0.032). The miR-29a-3p expression level in PD patients was significantly lower than ET patients (*p* = 0.035), but higher than MSA patients (*p* < 0.0001). The diagnostic efficacy reached a little higher when the combination of miR-29a-3p and miR-29c-3p.

**Conclusion:** The miRNA combination of salivary miR-29a-3p and miR-29c-3p has potential to be a diagnostic biomarker for idiopathic PD.

## Introduction

Diagnosis of PD is currently based on clinical symptoms of bradykinesia, rest tremor, rigidity, and beneficial response to dopaminergic therapy. The accuracy of clinical diagnosis is currently inadequate (79.6–83.9% by movement disorder experts) and presents difficulties in differentiating idiopathic PD from other forms of parkinsonism (1). Although various studies have identified potential PD biomarkers, currently only a few have been tested in clinical practice. Furthermore, although some diagnostic biomarkers can distinguish PD patients from HCs, their specificity in distinguishing non-PD neurological disease controls, especially parkinsonian syndrome, is wanting (2). Reliable and accurate biomarkers for PD are urgently needed to improve the accuracy of clinical diagnosis.

MiRNAs are conserved, small non-coding RNA molecules that can serve as posttranscriptional regulators of gene expression. MiRNAs may contribute to PD pathogenesis via the key processes, including apoptosis, neuroinflammation, mitochondrial dysfunction and proteasomal degradation (3). Some specific miRNAs have been shown to regulate PD-related genes and target directly or indirectly α-synuclein accumulation (4). MiRNAs are relatively stable in body fluids and easily quantified by routine and fast laboratory methods (5). An increasing number of studies have evaluated differential miRNA expression in different peripheral fluids of PD patients including peripheral blood mononuclear cells, serum, plasma, and cerebral spinal fluid (CSF) (3). Recent meta-analysis has identified several miRNAs in brain and blood tissues with highly significant differential expression in PD, indicating the potential of miRNAs as biomarkers for diagnosis (6). However, only a few studies have evaluated the disease specificity of miRNAs among PD, MSA and ET. Serum miR-7641, miR-191 and plasma miR-19b-3p may be useful for differentiating PD and multiple system atrophy (MSA) (7, 8). Plasma hsa-miR-4639-5p may discriminate between PD patients and ET patients (9). However, current characterizations of functional roles of miRNAs in PD are mostly at variance, and therefore remain challenging.

Saliva, a readily accessible body fluid, has emerged as an excellent source for biological components that could potentially be effective biomarkers (10). Salivary α-synuclein and DJ-1 protein display a reliable degree of consistency and validity as disease biomarkers (11). However, to date only two studies have described salivary miRNA expression differences between PD and HC (12, 13), and no study using salivary miRNA has provided a clear distinction of idiopathic PD from atypical parkinsonism or ET (14). In this study, we aimed to identify differentially expressed salivary miRNAs and validate a diagnostic potential for idiopathic PD. Furthermore, we also evaluate the specificity of candidate miRNAs for differentiating idiopathic PD from MSA and ET.

## Materials and Methods

### Subjects and Clinical Assessments

A total of 90 patients were consecutively recruited for this study. All were treated in outpatient and in ward at Peking University First Hospital from June 2020 to January 2021. Of these, 50 were diagnosed with idiopathic PD according to the Movement Disorders Society clinical diagnostic criteria (15), 20 were diagnosed with ET based on the consensus statement on the classification of tremor (16), and 20 were diagnosed with probable MSA according to consensus criteria (17). Eleven patients of the latter group had MSA of the parkinsonian form, and the other 9 had MSA of the cerebellar form. Thirty age-and sex-matched healthy controls (HCs) were also enrolled. Exclusion criteria included (1) severe periodontal disease, oral cancer, or gastroenteric tumor, (2) active systemic inflammatory diseases (for examples, tuberculosis, and rheumatoid arthritis.), (3) serious organic diseases of heart, liver, and kidney, and (4) drug or alcoholic abuse in the past year. The study was approved by the Ethical Committees of Peking University First Hospital and an informed written consent was taken from all the participants.

We obtained basic demographic and clinical data, including sex, age, disease duration, and current medication from each participant. For PD patients, the unified Parkinson's disease rating scale (UPDRS) and Hoehn and Yahr (H-Y) stage were used for the clinical evaluation of both motor and non-motor features during “off” state. The Mini-mental Status Examination (MMSE) was used for cognitive assessment. Levodopa equivalent daily dose (LEDD) was calculated by an established dose equivalence method (18).

### Study Design

**Figure 1:**
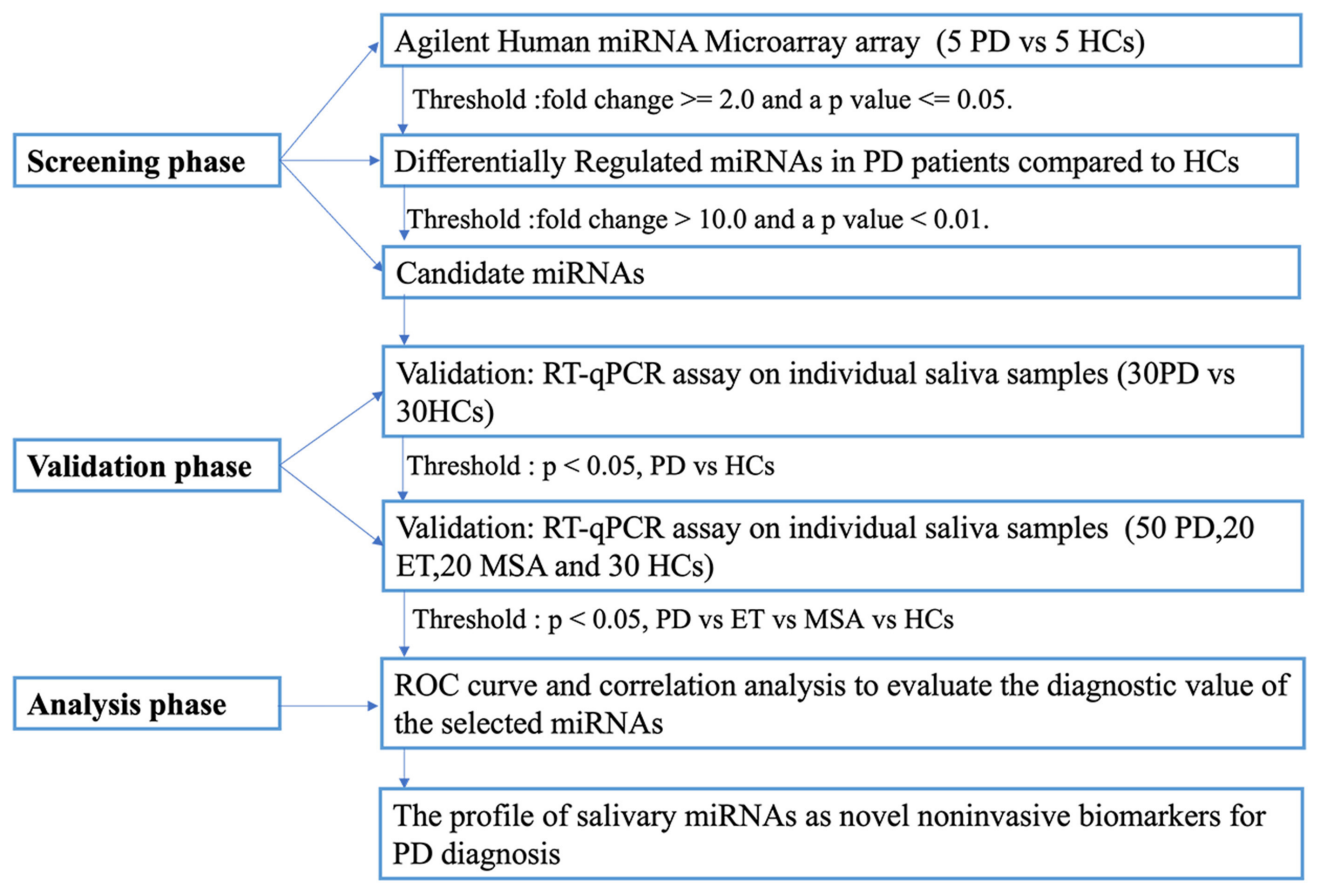
The overview of the experiment design.

A multiphase, case-control study design was used to identify salivary miRNA as diagnostic markers for PD (Figure 1).

### Saliva Collection

Before saliva collection, all patients and HCs were required to avoid eating or smoking for at least 2 h. Samples of 1–3 mL were collected and stored at 4°C for a maximum of 3 h before further processing. Samples with traces of blood were discarded. Samples were centrifuged at 12,000 ×g at 4°C for 20 min, and supernatants were transferred to 1.5 mL centrifuge tubes and stored at −80°C until RNA extraction.

### Microarray Analysis

Five randomly selected samples of both PD and HC were analyzed by OE Biotechnology Co., Ltd. (Shanghai, China) using the Agilent Human miRNA microarray kit, Release 21.0,8 × 60K. This microarray contains 2,570 probes for mature miRNA. Differentially expressed miRNAs were then identified through fold change analysis and student's *t*-test. Threshold criteria were set as follows: threshold set for up- and down-regulated genes was a fold change ≥2.0 and a *P* value ≤ 0.05. Target genes of differentially expressed miRNAs were the intersection predicted with two databases (miRDB, miRWalk). Kyoto Encyclopedia of Genes and Genomes (KEGG) molecular pathways and gene ontology (GO) analysis were applied to determine the roles of these target genes. Hierarchical Clustering was performed to show the distinguishable miRNAs expression pattern among samples.

### Quantification of miRNA Expression Levels

Total RNA from participant saliva was extracted in Trizol reagent (Life Technologies, Waltham, MA, USA) according to the instructions of the manufacturer as previously described (12). The miRNA quality and quantity were further confirmed using the Agilent 2100 bioanalyzer (Agilent Technologies). Total miRNA was reverse transcribed using the miRcute Plus miRNA First-Strand cDNA Kit (TIANGEN, Beijing, China). The reverse transcription protocol was as follows: 60 min at 42°C for polyadenylation and reverse transcription reaction and 3 min for enzyme inactivation reaction at 95°C. The obtained cDNA was used immediately for PCR or stored at −80°C. The Applied Biosystems 7500 Fast Real-Time PCR System (Applied Biosystems, USA) was used to quantify miRNA with miRcute Plus miRNA qPCR Kit (TIANGEN, Beijing, China) via quantitative real-time polymerase chain reaction (RT-qPCR). The reaction mixtures were incubated at 95°C for 5 min, followed by 45 cycles of 94°C for 20 s, 60°C for 34 s. RT-PCR was performed in triplicate on a Lightcycler 96. For each panel, miRNAs were normalized by U6 snRNA. The miRNAs primers sequences were shown in Supplementary Table S1. Expression levels were calculated using the 2^−ΔΔCt^ method and only miRNAs with cycle threshold (Ct) values <35 was included in the analyses.

### Statistical Analysis

Statistical analysis and graphing were performed using software SPSS 25.0 (IBM Corp., Armonk, NY, USA) and GraphPad Prism 8.0 software (San Diego, CA, USA). The relative expression level of miRNAs in patients and HCs were expressed as the median and compared using the Mann-Whitney test. After the expression levels were log transformed for normal distribution, data were presented as mean ±standard deviation and compared using student's *t*-test. Analysis of covariance was used to assess the difference in mean values of age, disease duration, and salivary miRNA levels between PD, ET, MSA and HC groups. Receiver operating characteristic (ROC) curve analysis and areas under curves (AUC) was performed to evaluate the miRNAs' diagnostic usefulness. The optimal cutoff points were established based on the maximum Youden's index. Sensitivity and specificity were also analyzed. For correlations analysis, Spearman's test was used. Two-sided tests were used, and *p* < 0.05 was considered significant.

## Results

### Clinical Data of Patients and Controls

The demographic and clinical characteristics of the PD, ET, MSA patients and healthy controls are summarized in Table 1. There was no significant difference among patients and HCs for the distribution of gender and age (*p* = 0.702 and *p* = 0.316, respectively). The mean disease duration of patients with ET was significantly longer than PD patients and MSA patients (*p* < 0.001). There was no significant difference between PD and MSA patients (*p* = 0.282). The distribution of the 50 PD patients according to H-Y stage was: 14 patients (28.0%) in stage 1, 17 (34.0%) in stage 2, 4 (8.0%) in stage 2.5,14 (28.0%) in stage 3 and 1 (2.0%) in stage 4.

**Table 1:** Demographic of patients and controls.

**Parameters**	**PD (*n* = 50)**	**MSA (*n* = 20)**	**ET (*n* = 20)**	**HC (*n* = 30)**	***P*** **-value**
Male, *n* (%)	19 (38.0)	6 (30.0)	8 (40.0)	14 (46.7)	0.702
Age	63.62 ± 11.65	63.00 ± 7.74	64.70 ± 9.07	59.67 ± 11.18	0.316
Disease duration	3.70 ± 2.97	2.78 ± 1.52	8.55 ± 4.76	N/A	0.000
H-Y stage	2.08 ± 0.82	N/A	N/A	N/A	N/A
UPDRS III score	24.16 ± 10.97	N/A	N/A	N/A	N/A
LEDD (mg)	325.00 ± 297.07	N/A	N/A	N/A	N/A

*Data were expressed as numbers with percentages in parentheses or as mean ± standard deviation. Analysis of covariance followed by Bonferroni post-hoc test was used. Age and Disease duration is counted in years. HC, healthy control; PD, Parkinson's disease; MSA, multiple system atrophy. ET, essential tremor. H-Y, Hoehn and Yahr; UPDRS, unified Parkinson's disease rating scale; LEDD, levodopa equivalent daily dose. N/A, not applicable*.

### Identification of Differentially Expressed Salivary miRNAs in PD Patients by miRNA Microarray Analysis

**Figure 2:**
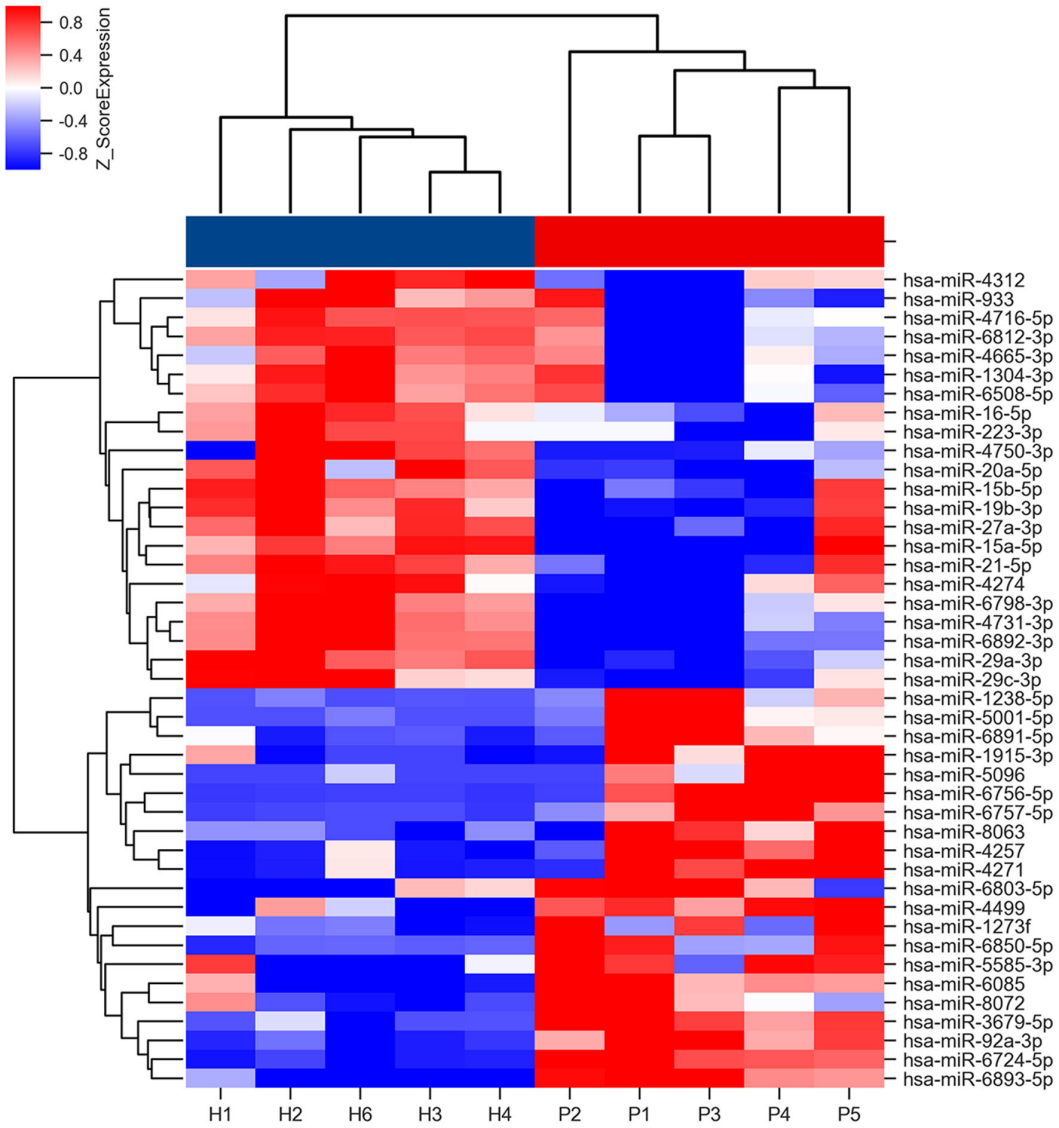
Heat map of miRNA microarray expression data of PD patients and HCs. Rows: miRNAs. Columns: HC and PD samples. Red, white, and blue indicate the up-regulation, unchanged expression, and down-regulation of miRNAs, respectively. The bar represents the scale of relative miRNA expression levels based on Log2 value. Student's *t*-test was used for comparisons between PD and HC groups.

The miRNA microarray comparisons identified 21 miRNAs that were upregulated, and 22 that were downregulated in the saliva of the patients with PD (Supplementary Table S2). The heat map of these differentially expressed miRNAs is shown in Figure 2. The top GO slim categories controlled by the union of these deregulated miRNAs was “ubiquitin protein ligase activity” in the molecular function (Supplementary Figure S1). The top KEGG categories for all deregulated miRNAs included Longevity regulating pathway, Axon guidance, Thyroid hormone signaling pathway, and ErbB signaling pathway (Supplementary Figure S2).

### The Validation of Candidate miRNAs in PD Patients and HCs by RT-qPCR

**Figure 3:**
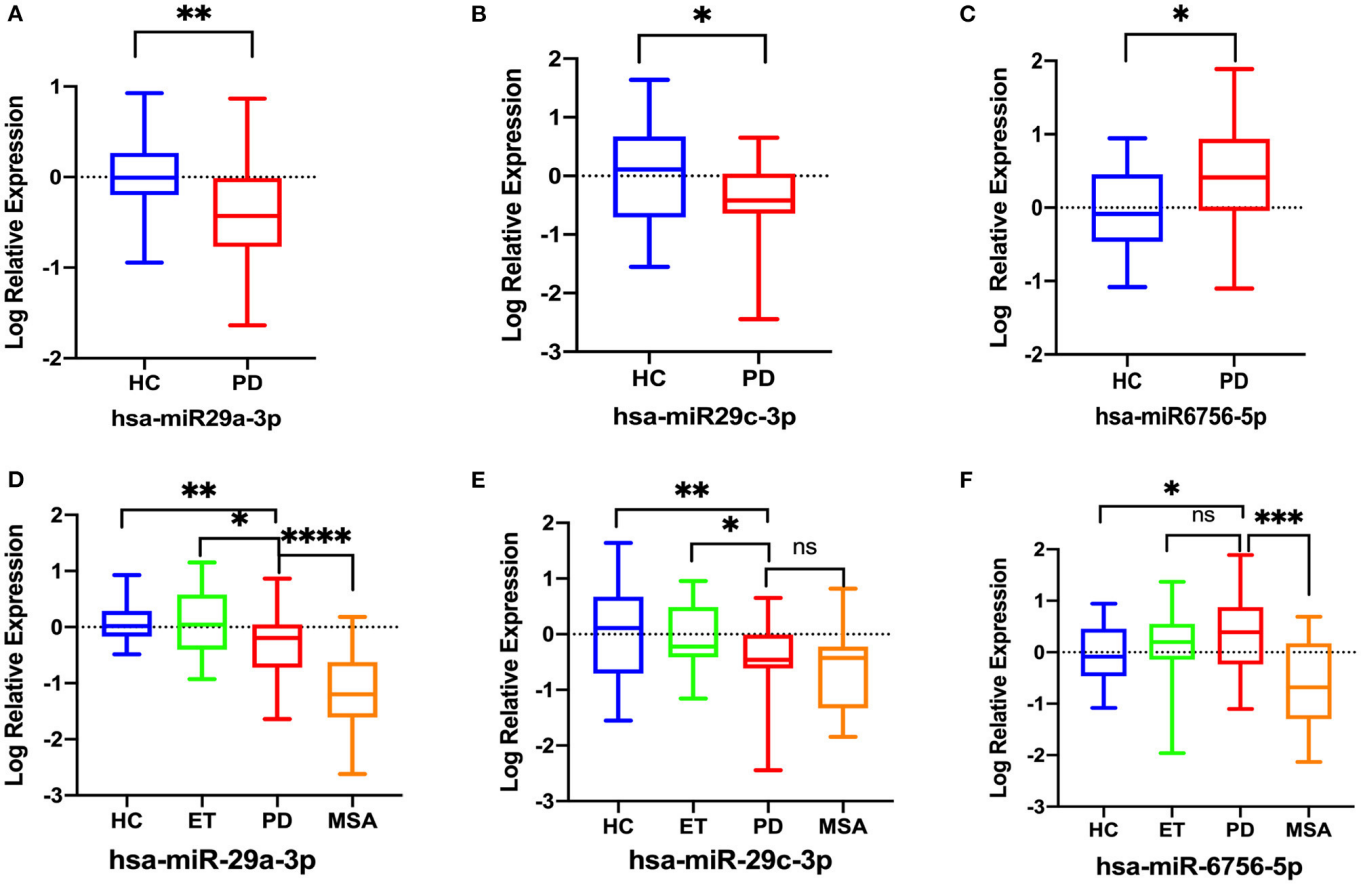
Comparison of the relative expression of salivary miRNAs in 30 PD patients and 30 HCs. Data were normalized via log transformation and reported for miR-29a-3p **(A)**, miR-29c-3p **(B)**, miR-6756-5p **(C)**. Comparison of the relative expression of salivary log-transformed salivary miRNAs between the PD, ET, MSA patients and HCs. **(D)** miR-29a-3p, **(E)** miR-29c-3p, **(F)** miR-6756-5p; Differences were analyzed by Student's *t*-test **(A–C)** and analysis of covariance followed by Bonferroni *post-hoc* test **(D–F)**. *P < 0.05; **P < 0.01, ***P < 0.001, ****P < 0.0001, ns, not significant.

In the validation phase, RT-qPCR was used to validate eight candidate miRNAs (miR-29a-3p, miR-29c-3p, miR-6085, miR-6724-5p, miR-6893-5p, miR-6756-5p, miR-6892-3p, and miR-4731-3p) in 30 PD patients and 30 HCs. Data revealed that three of eight miRNAs were deregulated in PD patients. MiR-29a-3p (*p* = 0.004) and miR-29c-3p (*p* = 0.027) were significantly downregulated, whereas miR-6756-5p (*p* = 0.032) was significantly upregulated in the PD patients when compared with HCs. The remaining five miRNAs displayed no significant expression level difference between the two groups (all *p* > 0.05) (Supplementary Table S3). Log-transformed miR-29a-3p and miR-29c-3p levels remained significantly lower, and log-transformed miR-6756-5p remained significantly higher in the PD patients compared with HCs (Figures 3A–C). However, log-transformed expression levels of miR-6085, miR-6724-5p, miR-6892-3p, and miR-4731-3p were not significantly different in PD patients when compared with HCs (Supplementary Table S4).

### The Expression of Candidate miRNAs Among PD, ET, MSA Patients and HCs by RT-qPCR

Then, RT-qPCR was used to investigate the expression of the three significantly deregulated miRNAs in all recruited subjects: 50 PD, 20 ET, 20 MSA patients, and 30 HCs. Analysis of covariance followed by Bonferroni *post-hoc* test was used for multiple comparisons. The log-transformed miR-29a-3p were significantly downregulated in PD patients compared to HCs (*p* = 0.002) and ET patients (*p* = 0.035), but higher than in MSA patients (*p* < 0.0001) (Figure 3D). The log-transformed miR-29c-3p were significantly downregulated in PD patients compared to HCs (*p* = 0.002) and ET patients (*p* = 0.018), whereas there were no significant differences between PD patients and MSA patients (*p* = 0.418) (Figure 3E). The log-transformed miR-6756-5p were significantly upregulated in PD patients when compared with HCs (*p* = 0.028) and MSA patients (*p* = 0.0004), whereas there were no significant differences between PD patients and ET patients (*p* = 0.842) (Figure 3F; Supplementary Table S5).

### ROC Curve Analysis

**Figure 4:**
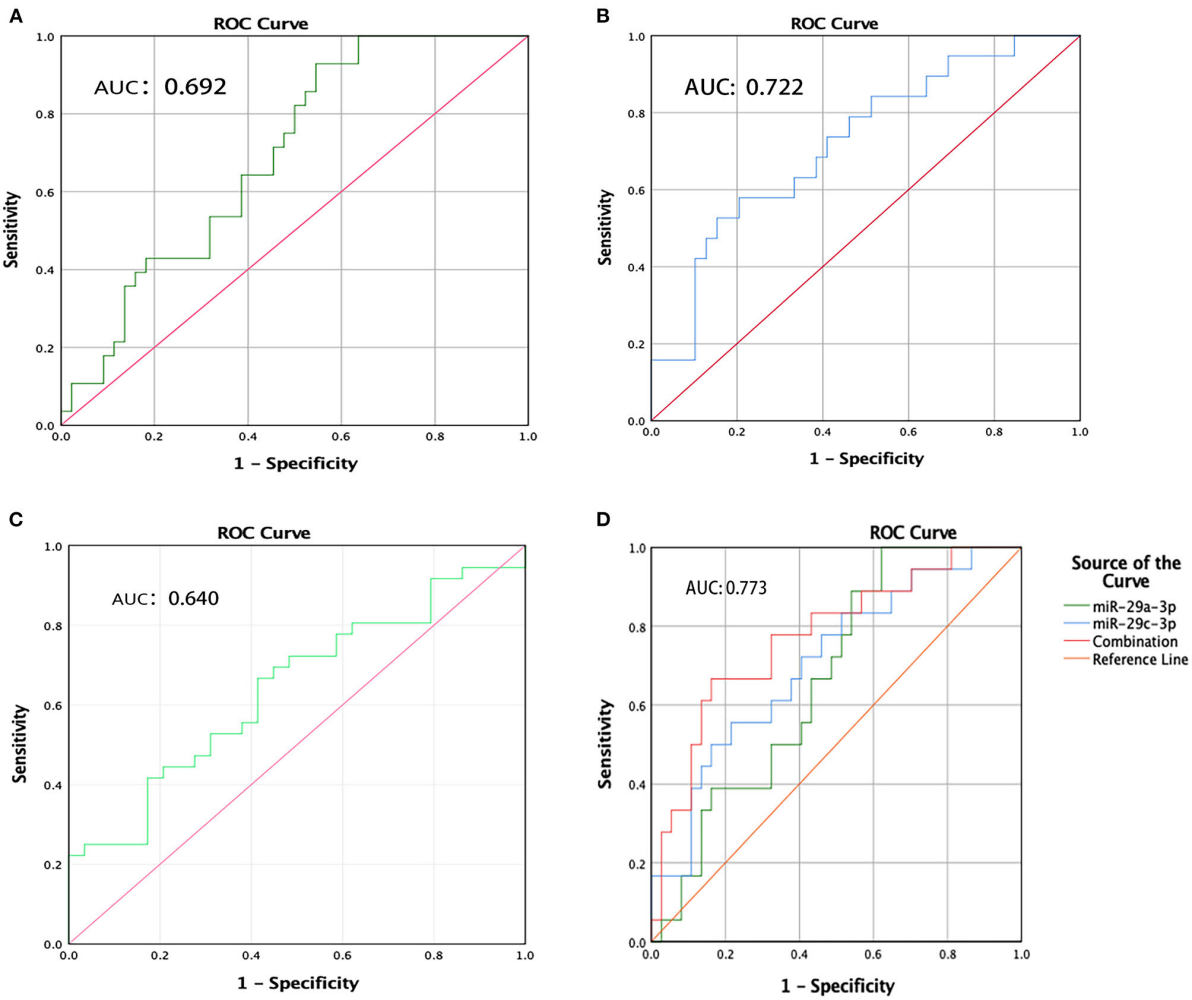
ROC curve analysis of miR-29a-3p, miR-29c-3p and miR-6756-5p levels in PD patients. **(A)** ROC curves of miR-29a-3p. **(B)** ROC curves of miR-29c-3p. **(C)** ROC curves of miR-6756-5p. **(D)** ROC curves of combination miR-29a-3p and miR-29c-3p.

To evaluate the usefulness of the three deregulated miRNAs for detecting PD, ROC curves were constructed. The corresponding AUCs were as follows: miR-29a-3p, 0.692 (95% CI, 0.573–0.812); miR-29c-3p, 0.722 (95% CI, 0.583–0.861); and miR-6756-5p, 0.640 (95% CI, 0.505– 0.774) (Figures 4A–C). At the arbitrary threshold of 0.56, miR-29a-3p sensitivity was 79.3% and specificity was 51.2%; miR-29c-3p sensitivity was 65.4% and specificity was 70.6% at the arbitrary threshold of 0.48; and miR-6756-5p sensitivity was 66.7% and specificity was 58.6% at the arbitrary threshold of 2.38, relative to HCs. We further evaluated the diagnostic value of the combination of miR-29a-3p and miR-29c-3p. The diagnostic sensitivity and specificity were 66.7 and 83.8%, respectively, and the AUC was 0.773 (95% CI, 0.639–0.908) (Figure 4D). To evaluate the predictive value of miR-29a-3p for distinguishing PD from MSA patients, ROC curve analysis was performed. The AUC was 0.894 (0.812, 0.975) at the arbitrary threshold of 0.18, the sensitivity was 65.0%, and specificity was 90.0%. The results suggest that salivary miR-29a-3p has better specificity in distinguishing MSA.

### Association Between miRNA Levels and Clinical Features

Spearman correlation analysis was used to correlate the expression levels of the three deregulated miRNAs with clinical features. We found that both the expression level of hsa-miR-29a-3p and hsa-miR-29c-3p did not correlate with the age, disease duration, UPDRS III score, MMSE score, or LEDD. The expression level of hsa-miR-6756-5p was also uncorrelated with age, disease duration, UPDRS III score, MMSE score, or LEDD of PD patients (Table 2).

**Table 2:** Association between miRNAs expression levels and clinical features.

**Clinical features**	**Age rho (*p*)**	**Duration rho (*p*)**	**UPDRSIII rho (*p*)**	**MMSE rho (*p*)**	**LEDD rho (*p*)**
miR-29a-3p	0.279 (0.06)	0.268 (0.07)	0.234 (0.118)	−0.07 (0.665)	0.295 (0.127)
miR-29c-3p	0.156 (0.395)	−0.121 (0.511)	−0.073 (0.693)	0.008 (0.964)	−0.185 (0.447)
miR-6756-5p	0.213 (0.176)	−0.178 (0.254)	−0.243 (0.121)	−0.095 (0.552)	0.041 (0.848)

*Rho, correlation coefficient; Spearman's test was used. UPDRS, Unified Parkinson's disease rating scale; MMSE, Mini-mental Status Examination; LEDD, Levodopa equivalent daily dose*.

## Discussion

The identification of premotor biomarkers is not only crucial to the early diagnosis of PD but is also helpful in the development of effective neuroprotection. To date, symptom markers, imaging and biological markers and genetic biomarkers have been widely investigated. Among these biomarkers, the highest diagnostically valuable ones include Rapid eye movement sleep behavior disorder (RBD) and dopaminergic imaging (19). However, increasing evidences have shown that RBD is also found in non-synucleinopathy neurodegenerative diseases (20). The abundance of gut microbiota in PD patients is significantly altered, which may be used as potential biomarkers or therapeutic targets (21). Specific PD gene, SYNJ1 is useful to diagnose familial PD and may be promising markers in discriminating PD from HCs and Parkinson's Plus Syndrome (22). However, only a few have been tested in clinical practice (2). Non-coding RNAs including long ncRNAs, circular RNAs, miRNAs and tRNA-derived fragments play important regulatory roles in neurodegenerative diseases, which may provide a great opportunity for non-invasive diagnosis assessment (23–25). To our knowledge, the current study is the first to document the global miRNA expressions in saliva of PD patients using genome-wide array analysis and identity differentially expressed miRNAs as potential biomarkers to discriminate idiopathic PD patients from HCs. Bioinformatics analysis of deregulated miRNA targets revealed that ubiquitin protein ligase activity was significant in molecular function. Early studies implicated that deficient ubiquitin-proteasome systems (UPS) played a critical role in the molecular pathogenic mechanisms of PD (26). A recent study showed the activation of UPS, via small molecular UPS enhancers, may be a therapeutic strategy for intervention against PD (27). The top pathways included axon guidance, thyroid hormone signaling, and ErbB signaling pathways, all of which had been previously associated with PD. Similar results were also found in plasma brain-enriched miRNAs of PD (5). Therefore, these results indicate that salivary miRNA deregulation patterns correlate with PD pathogenesis.

Among 43 differentially expressed miRNAs, ten have been previously reported as significantly deregulated in PD brain tissues, blood, CSF, or models. MiR-223 has been identified as significantly deregulated in PD saliva (13). MiR-15b-5p, miR-19b-5p, miR-29a-3p and miR-29c-3p were down-regulated, miR-27a-3p was up-regulated in PD blood and miR-92a-3p showed significant expression differences in PD brain tissue (3, 28). Previous studies have confirmed that miR-16 and miR-21 indirectly impact on α-synuclein aggregation in PD models (4). MiR-15a-5p has been found to be differentially expressed in PD by analyzing publicly available microarray datasets (29). Beyond these, our study identified 33 novel miRNAs differentially expressed in PD saliva. However, it is exceedingly difficult to validate all the candidate miRNAs. One well-executed study, based on a hypothesis from murine models, found miRNA-153 and miRNA-223 alterations between PD patients and HCs (13). The other study in PD saliva, investigated miRNA-874 and miRNA-145-3p (12). In our study, we did not identify the previously reported miRNAs except miRNA-223 in the screening stage. Overall, these results have been inconsistent due to small sample size, different study designs and analytical challenges. As the number of individuals in the screening phase is small and miRNA microarray data have reproducibility and replicability issues, some potential miRNAs might have been filtered out. Recently, mounting evidence supports miRNAs have been revealed to be regulated by the PD risk genes and may contribute to PD through a direct regulation on the mitochondrial and immune pathways (3). Besides, several miRNAs are critical players for α-synuclein protein accumulation and neuroinflammation (30). Therefore, we speculate there would be a series of significantly differentially expressed miRNAs in PD saliva. Future studies will need to integrate the data across all the reports to identify steadily deregulated miRNAs.

Using RT-qPCR, our study further found that salivary miR-29a-3p and miR-29c-3p were significantly downregulated in PD patients. In addition, previous animal models have demonstrated that miR-29a down-regulation correlates with cell death in the brain (31). Additionally, miR-29a targets the voltage dependent anion channel1, and plays important roles in the pathologies of PD by regulating apoptosis (31, 32). A recent study found that overexpression of miR-29c could attenuate dopaminergic neuron loss and α-synuclein accumulation in substantia nigra pars compacta of PD mice (33). Our study also identifies one novel differentially expressed miRNA (miR-6756-5p) which is significantly upregulated in PD saliva. Although the role of this miRNA in the pathogenesis of PD is unclear, the downstream targeting mRNA of miR-6756-5p includes ATG9A (miRTarBase) and glucocerebrosidase mRNAs (TargetScanHuman 7.2), which are risk factors for PD. Specific miRNAs may be crucial to PD pathogenesis through its effects on core neurodegenerative processes such as oxidative stress, inflammation, and apoptosis. By extension, understanding miRNA-regulated molecular mechanisms can develop innovative targeted therapy.

It is worth noting that the sensitivity and specificity of using a single miRNA are suboptimal in both our study and previous reports. Our results showed the AUCs of the three deregulated miRNAs were 0.692, 0.722, and 0.640, respectively. In the other two studies, the AUCs of four different salivary miRNAs for distinguishing PD from HC were 0.707–0.790 (12, 13). However, the present study showed that the combination of miR-29a-3p and miR-29c-3p could enhance the specificity over a single biomarker and up to 83.8%. Therefore, the combination of miRNAs might achieve a higher predictive biomarker performance. The identification of biomarkers for identifying PD from MSA is strongly desired. “hot cross bun” sign grade may be a useful imaging indicator for the MSA-C subtype, but not MSA-P cases (34). We found salivary miR-29a-3p has adequate specificity for differentiating idiopathic PD from MSA and ET. Previous studies indicated that serum miRNA-7641 and miRNA-191 could differentiate MSA from PD, and that plasma miR-4639-5p had adequate sensitivity and specificity to discriminate between PD and ET patients (8, 9). Despite these recognized limitations of diagnostic accuracy, salivary miRNA has promising diagnostic value.

We found the expression of all three deregulated miRNAs had no clear correlation to age, gender, disease course, or LEDD. Therefore, the deregulated miRNAs expression in PD patients is stable, with little variation between sex, age, disease course or anti-parkinsonian medications. One Chinese study illustrated that the expression levels of miR-29a and miR-29c decreased more in the PD patients with dementia compared with PD patients with normal cognition (35). This discrepancy may be explained by the fact that our study has not recruited PD patients with dementia.

There were several limitations to our study. First, the current sample population represented a single site, and the sample sizes were relatively small. Second, tau protein disease should also be analyzed to determine differential diagnostic value. Third, future studies are needed to explore underlying mechanisms.

## Conclusion

In summary, we found 34 differential miRNAs in PD saliva which may be implicated in the pathogenesis of the disease and in molecular functions such as ubiquitin protein ligase activity. We identified three differentially expressed miRNAs (miR-29a-3p, miR-29c-3p, and miR-6756-5p). The combination of salivary miR-29a-3p and miR-29c-3p has potential to be a non-invasive diagnostic biomarker for idiopathic PD, and miR-29a-3p may be useful for differentiating PD from ET and MSA. Further studies validating and expanding our findings in larger PD cohorts are warranted.

## Data Availability Statement

The datasets presented in this study can be found in online repositories. The name of the repository and accession number can be found below: The European Molecular Biology Laboratory's European Bioinformatics Institute (EMBL-EBI) ArrayExpress, https://www.ebi.ac.uk/arrayexpress/, E-MTAB-10779.

## Ethics Statement

The studies involving human participants were reviewed and approved by Ethical Committees of Peking University First Hospital. The patients/participants provided their written informed consent to participate in this study.

## Author Contributions

YJ, JC, JD, YY, and ZW contributed to the study concept and design. YJ, JC, YS, FL, LW, WS, and ZW contributed to clinical diagnosis and data collection. YJ and JD carried out the experiments and acquisition of the data. YJ and JC completed the statistical analysis and manuscript editing. JD, YY, and ZW contributed to the critical revision of the manuscript. All authors contributed to the article and approved the submitted version.

## Funding

This work was funded by the UULM-PUHSC-Joint-Center, Grant/Award Number: PKU2017ZC001-4; National Natural Science Foundation of China (Nos. 81571219, 82071409, and U20A20356 to ZW) and Peking University Medicine Fund of Fostering Young Scholars' Scientific & Technological Innovation (BMU2021PY003 to JD).

## Conflict of Interest

The authors declare that the research was conducted in the absence of any commercial or financial relationships that could be construed as a potential conflict of interest.

## Publisher's Note

All claims expressed in this article are solely those of the authors and do not necessarily represent those of their affiliated organizations, or those of the publisher, the editors and the reviewers. Any product that may be evaluated in this article, or claim that may be made by its manufacturer, is not guaranteed or endorsed by the publisher.
