# Supplementary material for: Profiling of Differentially Expressed MicroRNAs in Saliva of Parkinson's Disease Patients
Source: Front Neurol. 2021 Nov 26;12:738530. doi: 10.3389/fneur.2021.738530 (PMC8660675; doi:10.3389/fneur.2021.738530)
Supplement: Supplementary file 1 [file Table_1.DOCX]

**Table S1**

**Table S1 The primer sequences used in RT-qPCR（5'-3'）**

| miRNAs | The primer sequences（5'-3'） |
| --- | --- |
| hsa-miR-29a-3p | GAGGCTGAGTCGTAGCACCATC |
| hsa-miR-4731-3p | ACAAGTGGCCCCCAACACT |
| hsa-miR-29c-3p | GTATCGGCTGTAGCACCATTTG |
| hsa-miR-6892-3p | CCTCTCCCACCCCTTGCAG |
| hsa-miR-6893-5p | CAGGCAGGTGTAGGGTGGAG |
| hsa-miR-6724-5p | ATATACTGGGCCCGCGGC |
| hsa-miR-6756-5p | GGGCTGGAGGTGGGGCT |
| hsa-miR-6085 | AGGGGCTGGGGGAGCAC |
| hsa-miR-92a-3p | TATTGCACTTGTCTCGGCCTGT |
| U6-1-F | ATTGGAACGATACAGAGAAGATT |
| U6-1-R | GGAACGCTTCACGAATTTG |
