# Supplementary material for: Profiling of Differentially Expressed MicroRNAs in Saliva of Parkinson's Disease Patients
Source: Front Neurol. 2021 Nov 26;12:738530. doi: 10.3389/fneur.2021.738530 (PMC8660675; doi:10.3389/fneur.2021.738530)
Supplement: Supplementary file 2 [file Table_2.DOCX]

**Table S2**

**Table S2 Differentially expressed miRNAs**

| Probel ID | FC (PD vs HC) | p Value | Regulation |
| --- | --- | --- | --- |
| hsa-miR-6085 | 17.5580499445795 | 0.00314855880572496 | up |
| hsa-miR-6724-5p | 16.8611873476143 | 8.60360361458365E-06 | up |
| hsa-miR-6893-5p | 15.5412224076129 | 7.99917086873048E-05 | up |
| hsa-miR-5585-3p | 13.4306331748519 | 0.0460166699195513 | up |
| hsa-miR-5001-5p | 12.9803920882534 | 0.0297564593917421 | up |
| hsa-miR-6756-5p | 11.8013409318772 | 0.00504294941421318 | up |
| hsa-miR-6850-5p | 8.32039558134002 | 0.0255981774416315 | up |
| hsa-miR-3679-5p | 8.14070561234214 | 0.001077692215561 | up |
| hsa-miR-6891-5p | 7.09298350551077 | 0.0372030486721558 | up |
| hsa-miR-6803-5p | 6.81304602452523 | 0.0474090753966719 | up |
| hsa-miR-1915-3p | 6.71170103425176 | 0.0421255532447667 | up |
| hsa-miR-1238-5p | 6.63254823741044 | 0.0334335305085311 | up |
| hsa-miR-4257 | 6.47061183993374 | 0.00861806682125627 | up |
| hsa-miR-4271 | 5.66880851504878 | 0.0140935035858403 | up |
| hsa-miR-4499 | 5.65255611706216 | 0.00421749506756978 | up |
| hsa-miR-8072 | 4.8718821596685 | 0.0395288688054937 | up |
| hsa-miR-92a-3p | 4.61781989846612 | 0.000241435409914111 | up |
| hsa-miR-8063 | 4.2250190067006 | 0.0465891454989222 | up |
| hsa-miR-6757-5p | 3.88209837956874 | 0.00878463264040963 | up |
| hsa-miR-1273f | 2.99079063108988 | 0.0427539710555112 | up |
| hsa-miR-5096 | 2.17030040814289 | 0.0394995798427327 | up |
| hsa-miR-29a-3p | -24.0139493677776 | 0.000226335938783858 | down |
| hsa-miR-29c-3p | -18.0267129075755 | 0.00256319863340588 | down |
| hsa-miR-27a-3p | -13.0455341192655 | 0.0130617481217392 | down |
| hsa-miR-19b-3p | -12.7486677788472 | 0.00925516438207115 | down |
| hsa-miR-6892-3p | -10.6728958410114 | 0.000175401085503156 | down |
| hsa-miR-4731-3p | -10.3408133804782 | 0.000621912081706014 | down |
| hsa-miR-6798-3p | -9.45878575248422 | 0.00364833168154392 | down |

Continued table S2 Differentially expressed miRNAs

| Probel ID | FC (PD vs HC) | p Value | Regulation |
| --- | --- | --- | --- |
| hsa-miR-20a-5p | -8.44844299142448 | 0.00270112132510904 | down |
| hsa-miR-15a-5p | -7.7709108510851 | 0.018506876976863 | down |
| hsa-miR-15b-5p | -7.38786804056135 | 0.0130436260813093 | down |
| hsa-miR-1304-3p | -7.34289400049935 | 0.0346938206397118 | down |
| hsa-miR-6508-5p | -7.02590269931775 | 0.0318241561767624 | down |
| hsa-miR-6812-3p | -6.47242681784396 | 0.0156304048210977 | down |
| hsa-miR-4750-3p | -6.15749817855108 | 0.0391392342397628 | down |
| hsa-miR-4312 | -6.11381104204698 | 0.0277360303223574 | down |
| hsa-miR-21-5p | -5.67451357520678 | 0.0137231488448776 | down |
| hsa-miR-933 | -4.61332936322914 | 0.0480995422071546 | down |
| hsa-miR-223-3p | -4.57506076051808 | 0.029255304437393 | down |
| hsa-miR-4274 | -4.5417409807779 | 0.0416386855646374 | down |
| hsa-miR-4716-5p | -4.35219397751765 | 0.0448839011311607 | down |
| hsa-miR-16-5p | -3.89898928435446 | 0.0325322623979838 | down |
| hsa-miR-4665-3p | -2.12401160318749 | 0.0467343473478443 | down |
