# Supplementary material for: Profiling of Differentially Expressed MicroRNAs in Saliva of Parkinson's Disease Patients
Source: Front Neurol. 2021 Nov 26;12:738530. doi: 10.3389/fneur.2021.738530 (PMC8660675; doi:10.3389/fneur.2021.738530)
Supplement: Supplementary file 3 [file Table_3.DOCX]

**Table S3**

**Table S3 Comparison of the relative expression of salivary miRNAs between the PD patients and HCs**

| Probel ID | HC (n=30) | PD (n=30) | p Value |
| --- | --- | --- | --- |
| hsa-miR-29a-3p | 1.0413320715 | 0.372982279 | **0.004** |
| hsa-miR-29c-3p | 1.236703796 | 0.419410045 | **0.027** |
| hsa-miR-6756-5p | 0.9599276725 | 2.450013337 | **0.032** |
| hsa-miR-6085 | 0.897508521 | 1.196121398 | 0.694 |
| hsa-miR-6892-3p | 1.664427245 | 1.58518521 | 0.661 |
| hsa-miR-6724-5p | 1.171505852 | 1.413963823 | 0.902 |
| hsa-miR-4731-3p | 0.670175171 | 1.665615062 | 0.749 |

Data were expressed as the median and compared using the Mann-Whitney test.
