# Supplementary material for: Profiling of Differentially Expressed MicroRNAs in Saliva of Parkinson's Disease Patients
Source: Front Neurol. 2021 Nov 26;12:738530. doi: 10.3389/fneur.2021.738530 (PMC8660675; doi:10.3389/fneur.2021.738530)
Supplement: Supplementary file 4 [file Table_4.DOCX]

**Table S4**

**Table S4 Comparison of the relative expression of salivary log-transformed miRNAs between the PD patients and HCs**

| Probel ID | HC (n=30) | PD (n=30) | p Value |
| --- | --- | --- | --- |
| hsa-miR-29a-3p | 0.00± 0.40 | -0.37±0.56 | **0.005** |
| hsa-miR-29c-3p | 0.05±0.88 | -0.44±0.64 | **0.023** |
| hsa-miR-6756-5p | -0.03±0.63 | 0.42±0.76 | **0.016** |
| hsa-miR-6085 | 0.00±0.65 | 0.12±0.77 | 0.518 |
| hsa-miR-6892-3p | 0.19±0.73 | 0.01±0.84 | 0.439 |
| hsa-miR-6724-5p | 0.09±0.75 | 0.21±0.74 | 0.584 |
| hsa-miR-4731-3p | -0.03±0.96 | 0.13±1.17 | 0.642 |

Data were presented as mean ±standard deviation and compared using student’s t-test.
