# Supplementary material for: Profiling of Differentially Expressed MicroRNAs in Saliva of Parkinson's Disease Patients
Source: Front Neurol. 2021 Nov 26;12:738530. doi: 10.3389/fneur.2021.738530 (PMC8660675; doi:10.3389/fneur.2021.738530)
Supplement: Supplementary file 5 [file Table_5.DOCX]

**Table S5**

**Table S5 Comparison of the relative expression of salivary log-transformed miRNAs between the PD, ET, MSA patients and HCs**

| Probel ID | HC (n=30) | PD (n=50) | ET (n=20) | MSA (n=20) | p Value |
| --- | --- | --- | --- | --- | --- |
| hsa-miR-29a-3p | 0.06± 0.33 | -0.32±0.57 | 0.07±0.62 | -1.18±0.72 | **0.000** |
| hsa-miR-29c-3p | 0.06±0.88 | -0.45±0.56 | -0.04±0.57 | -0.60±0.75 | **0.005** |
| hsa-miR-6756-5p | -0.03±0.63 | 0.37±0.79 | 0.08±0.76 | -0.62±0.87 | **0.000** |

Data were expressed as mean ± standard deviation and analysis of covariance followed by Bonferroni post-hoc test was used for multiple comparisons.
