# Supplementary material for: Profiling of Differentially Expressed MicroRNAs in Saliva of Parkinson's Disease Patients
Source: Front Neurol. 2021 Nov 26;12:738530. doi: 10.3389/fneur.2021.738530 (PMC8660675; doi:10.3389/fneur.2021.738530)
Supplement: Supplementary file 6 [file Data_Sheet_1.docx]

Supplementary Material


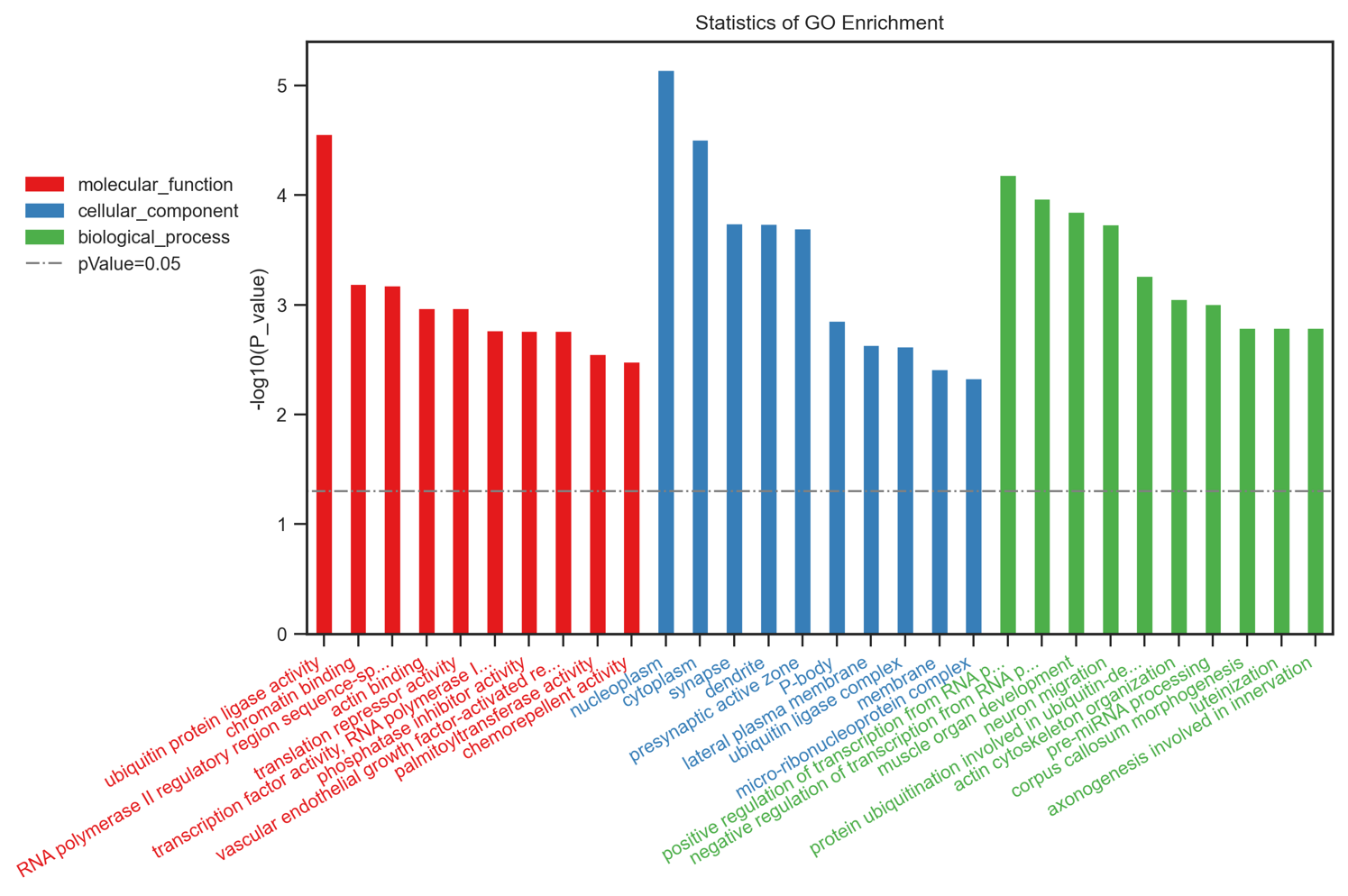


**Supplementary Figure 1.** Gene Ontology analysis of targets from differentially expressed miRNAs. The figure legends are required to have the same font as the main text, Data are shown as -log10 of P value for each term using Student's t-test. The Red bars showed molecular function terms; The Blue bars showed cellular component terms; The Green bars showed biological process.
