# Supplementary material for: Profiling of Differentially Expressed MicroRNAs in Saliva of Parkinson's Disease Patients
Source: Front Neurol. 2021 Nov 26;12:738530. doi: 10.3389/fneur.2021.738530 (PMC8660675; doi:10.3389/fneur.2021.738530)
Supplement: Supplementary file 7 [file Data_Sheet_2.docx]

Supplementary Material


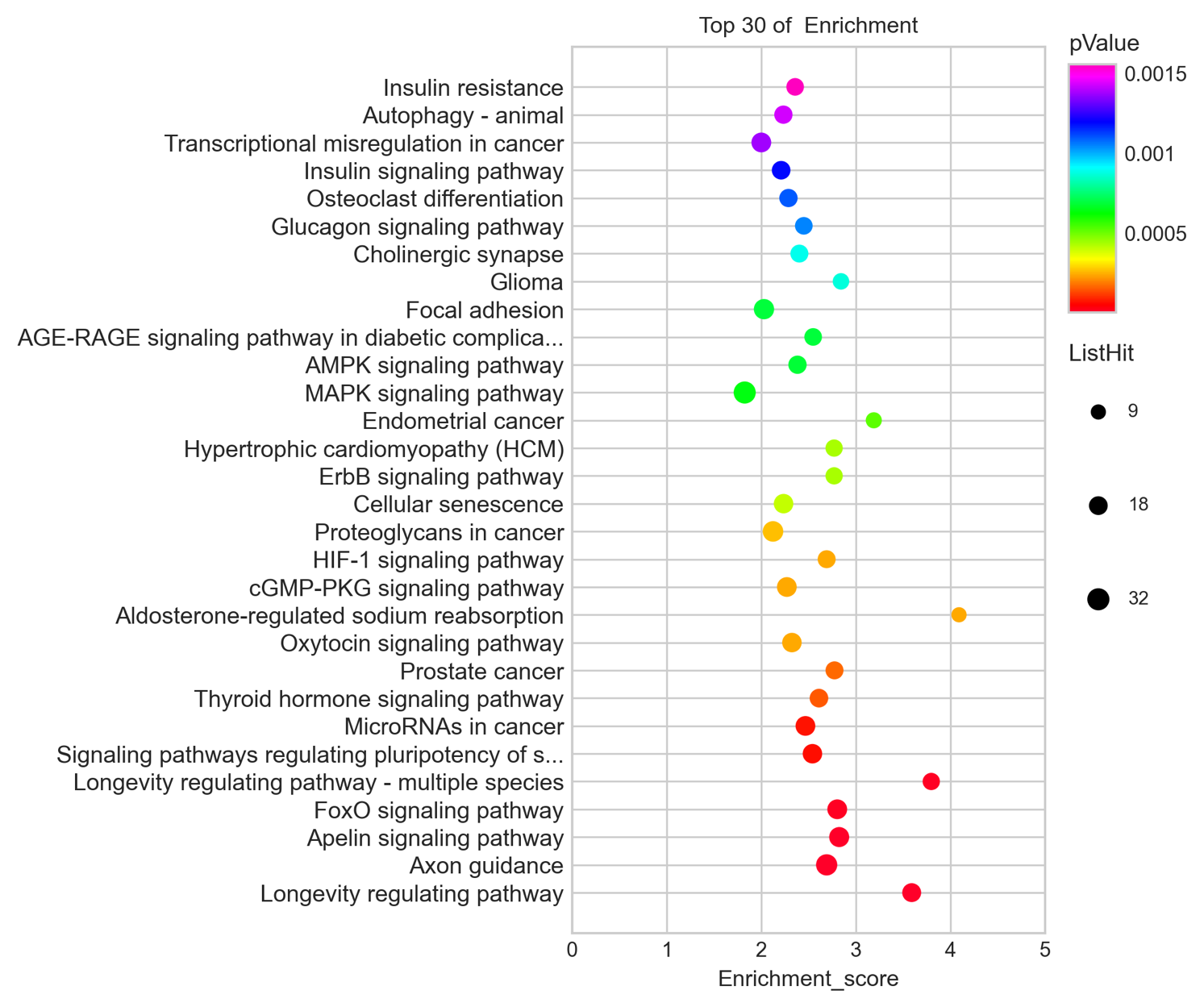


**Supplementary Figure 2.** The top 30 enriched Kyoto Encyclopedia of Genes and Genomes categories for all deregulated miRNAs. Rows: pathway. Columns: Enrichment score. The sizes of the dots represent the numbers of target genes that fall into the pathway. Dots represent enrichment significance with color coding: red indicates high enrichment and purple indicates low enrichment.
